# Adenoid cystic carcinoma of the esophagus: report of two cases and review of the Chinese literature

**DOI:** 10.1186/1746-1596-7-179

**Published:** 2012-12-13

**Authors:** Xu-feng Guo, Ten Mao, Zhi-tao Gu, Wen-tao Fang, Wen-hu Chen, Jin-chen Shao

**Affiliations:** 1 Department of Thoracic Surgery, Shanghai Chest Hospital, School of Medicine, Shanghai Jiao Tong University, Shanghai, 200030, China; 2 Department of Pathology, Shanghai Chest Hospital, School of Medicine, Shanghai Jiao Tong University, Shanghai, 200030, China

**Keywords:** Adenoid cystic carcinoma, Esophagus, Surgery

## Abstract

Squamous cell carcinoma is the major pathology type of esophageal cancer in China, where adenocarcinoma is rare and adenoid cystic carcinoma (ACC) is more rare comparing to the western countries. We report the surgical and pathologic findings of two cases of primary ACC of the esophagus, and review of the Chinese literature of this tumor.

**Virtual slides:**

The virtual slide(s) for this article can be found here: http://www.diagnosticpathology.diagnomx.eu/vs/1507582238843246

## Introduction

Adenoid cystic carcinoma (ACC) is not uncommon in the salivary glands and respiratory tract; however, it occurs extremely rarely in the esophagus, where its behavior is biologically aggressive [1]. It constitutes 0.1% of all esophageal malignancies, and only 60 cases have been reported so far in the literature [2]. Herein, we present 2 cases of ACC of the esophagus and review the only 13 other cases of ACC of the esophagus reported in China, in order to clarify the clinicopathological features of ACC of the esophagus.

## Case presentation

### Case 1

**Figure 1:**
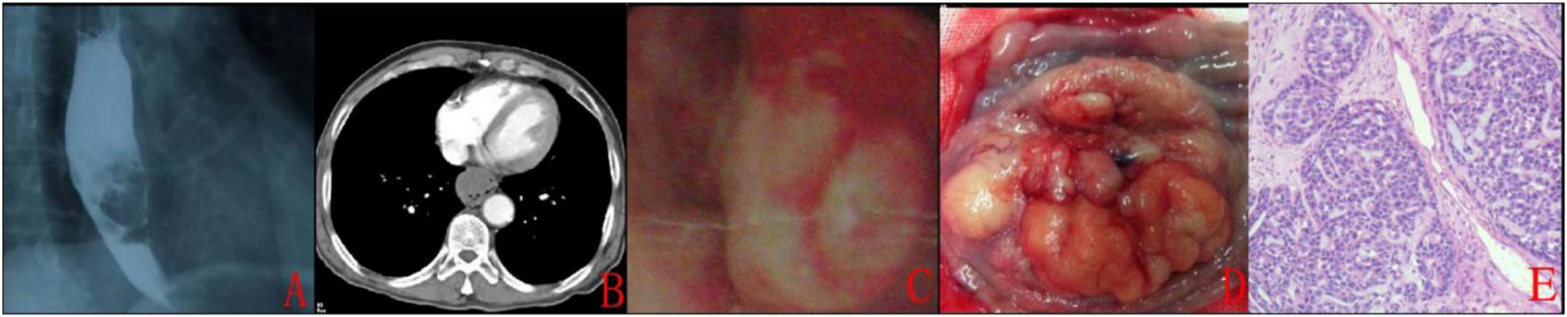
**(case 1)(A)Barium esophagram showing a protruding lesion in the middle third of the esophagus; (B) Computed tomography views of the case 1; (C) Endoscopic finding.** It showed a polypoid lesion at the mid-esophagus; (**D**) A cauliflower-like lesion; (**E**) It shows numerous enlarged hyperchromatic nuclei containing eosinophilic material (H&E stain, ×400).

A 70-year-old man was hospitalized with a 3-month history of progressive dysphagia. This patient’s occupation as a cook, he likes to eat hot, spicy foods, and no previous history of viral infection, there is no contact with any external toxic substances. However, this patient usually have reflux esophagitis. Barium esophagram revealed a protruding lesion of 5cm in length in the middle third of the esophagus (Figure 1A). Computed tomography of the chest showed a remarkable thickening of the mid-thoracic esophagus (Figure 1B). Esophagoscopy showed a cauliflower-like polypoid lesion (Figure 1C). A biopsy specimen suggested poorly differentiated squamous cell carcinoma, and a subtotal esophagectomy was performed (Ivor- Lewis). There was no evidence of direct invasion to the neighboring structures, lymphatic spread, or organ metastasis. The resected specimen consisted of 12 cm of the esophagus and 6cm of the upper portion of the stomach. A protuberant lobulated tumor, 6 × 3.8 × 2.5 cm in size (Figure 1D). Microscopic examination demonstrated an infiltrative malignant neoplasm composed of basaloid cells, exhibiting indistinct cell borders, scant amphophilic cytoplasm and enlarged hyperchromatic nuclei (Figure 1E). The tumor invaded the submucosa, but it had not metastasized to the lymph nodes (pT1bN0M0). Immunohistochemical studies revealed that tumor cells were stained immunohistochemically for CK, VIM, and Calponin protein. The patient was discharged from hospital 14 days after his operation, and no signs of recurrence have been detected in 5 months of follow up.

### Case 2

**Figure 2:**
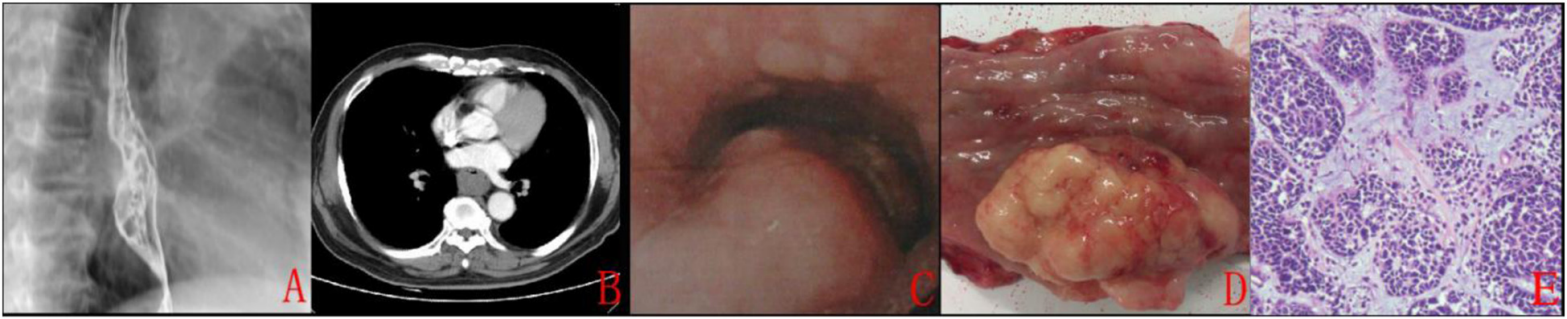
**(A) Barium esophagram; (B) Computed tomography; (C) Esophagoscopy; (D) specimen views; (E) It shows mixed cribriform and solid features (H&E stain, ×400.)**

A 65-year-old man who is a miner, no bad eating habits, but the long-term smoking, was admitted to our hospital with a 2-month history of dysphagia. Results of the examination of his chest and abdomen were unremarkable. Blood values were all normal on admission. The barium esophagogram showed a large protrusive smooth tumor, which was 5 cm in size in the mid-thoracic esophagus (Figure 2A). Computed tomography showed a remarkable thickening of the low-thoracic esophagus (Figure 2B). Esophagoscopy showed a cauliflower-like tumor with partially necrosis and located 30–36 cm from the incisors (Figure 2C). Multiple biopsies were taken which later showed evidence of malignant cells but of no specific type. Positron emission computed tomography showed no evidence of metastatic disease. The patient proceeded to a esophagectomy (Sweet) where intraoperatively tumor was confirmed with no evidence of local invasion.

The resected tumor was a cauliflower-like mass, measuring 5.5 × 4.2 × 3 cm in size, and pathologically invaded adventitia with no metastases to lymph nodes (Figure 2D). The tumor cells were small and darkly stained with scanty cytoplasm and vesicular nuclei. A cribriform pattern was seen, but there was a tendency for the formation of solid or basaloid areas with amorphous eosinophilic material and comedo necrosis (Figure 2E). Immunohistochemically, tumor cells expressed CK, VIM and P63 protein. Scattered cells expressed S-100 protein. The surgical resection margins were clear and all biopsies of lymph nodes were free of metastases. The pathologic diagnosis was reported as primary ACC of the esophagus (pT3N0M0).

The patient was diagnosed with anastomotic leak on post-operative day 10, then we gave him jejunal feeding tube guided by esophagoscopy. Barium esophagogram indicated the anastomotic leak was cured one month later. Nine months postoperatively, the computed tomography of the abdomen demonstrated liver metastasis.

## Discussion

ACC of the esophagus was rare, since Gregg [3] first reported it in 1954, only 15 cases including our 2 cases were reported in China. There were studies showed that people who with hiatal hernia, obesity (visceral fat), smoking or alcohol abuse as well, are more likely to presence of gastroesophageal reflux disease [4]. Then, infiltration of eosinophils into the esophagus could result to conditions such as eosinophilic esophagitis which may be the risk factor for development of ACC of the esophagus [5].

The clinical data of the total 15 cases are summarized in Table 1. The patients ranged in age from 42 to 70 years with an average age of 60.4 years, our patient being the oldest. The sex ratio was 11 men to 4 women, and the middle third of the esophagus was the most commonly affected region. Thus, the age, sex ratio, and the most commonly affected region in ACC seem to be similar to those of squamous cell carcinoma. The tumor appearance was protruded in 10 and ulcerative in 5 of the 15 cases in which the macroscopic appearance was reported. Normal esophageal mucosa overlying the tumor was reported in 3 of the 10 protruded cases. Patients with ACC of the esophagus generally present with dysphagia of several-month duration.

**Table 1:** Clinical data on the 15 cases of ACC of the esophagus reported in China

Ages	42-70 (60.4 ±6.6)
Sex(male : female)	11 : 4
Location	Cervical: 1 Up-thoracic: 0 Mid-thoracic: 8 Low-thoracic: 6
Macroscopic appearance	Protruding: 10 Ulcerative: 5
Biopsy result	
Adenoid cystic carcinoma	1
Squamous cell carcinoma	10
Leiomyomata	2
Unknown	1 (Misdiagnosed as upper esophageal sphincter achalasia)
Adenocarcinoma	1
Treatment	
Surgery	14
Chemoradiotherapy	1 (Preoperative brain metastasis)
Depth of invasion (Surgical cases)	
Lamina propria	2
Submucosa	7
Muscularis propria	1
Adventitia	4
Metastasis to lymph nodes (Surgical cases)	
LN positive	0
LN negtive	14

Biopsy of the tumor by preoperative endoscopy gave poor diagnostic results. In fact, only 1 of 14 such reported biopsies suggested ACC at that time. 10 biopsy specimens were misinterpreted as squamous cell carcinoma being the most frequent misdiagnosis, 2 cases were misdiagnosed as leiomyomata because of the normal esophageal mucosa overlying the tumor and 1 case was misdiagnosed as upper esophageal sphincter achalasia. Sweeney and Cooney reported [6] that one of the difficulties in diagnosing ACC of the esophagus from a biopsy specimen is related to the fact that small tissue samples may not display the characteristic architecture of the tumor. Esophageal adenoid cystic carcinoma diagnosed mainly rely on microscopic examination. Histologically, ACC of the esophagus show three different growth patterns similar to the salivary gland: cribriform, tubular and solid. The solid pattern appears to be associated with worse prognosis than the other two patterns [7]. The most commonly depth of invasion was submucosa (7/14) in our data. This may clarify the fact that ACC of the esophagus arise from the deep submucosa glands of the esophagus.

Because of the rarity of ACC of the esophagus and paucity of data in the literature, few treatment guidelines are available for esophageal ACC. The first choice for treatment of ACC of the esophagus is radical excision. Chemotherapy is not usually chosen due to a poor response rate [8-10]. Postoperative radiotherapy may help improvement of progressive dysphagia [11-13]. Unfortunately, we can not give a complete follow-up data in Chinese literature. Foreign learners reported that the prognosis of ACC of the esophagus was poor, with organ metastasis occurring more frequently than lymph node metastasis [14]. The 5-year survival rate is approximately 35%, but the long-term survival is poor. Eighty to 90% of patients die of this disease within 10–15 years [15].

## Conclusions

In a word, ACC of the esophagus is extremely rare. Its diagnosis must be based upon histopathological characteristics. Surgery is the first choice for resectable lesions.

### Consent

Written informed consent was obtained from the patient for publication of this Case Report and any accompanying images. A copy of the written consent is available for review by the Editor-in-Chief of this journal.

## Competing interests

The authors declare that they have no competing interests.

## Authors’ contributions

X-FG analyzed the data and wrote the manuscript as a major contributor. TM, Z-TG, W-TF and W-HC helped to revise the discussion section of this manuscript. J-CS conducted the pathological examination. All authors have read and approved the final manuscript.
